# Sustaining Programs: Lessons Learned from Former Federal Grantees

**DOI:** 10.1007/s10995-020-02878-7

**Published:** 2020-01-29

**Authors:** Alexandra Warner, Nicole Bennett, Subuhi Asheer, Julia Alamillo, Betsy Keating, Jean Knab

**Affiliations:** 1 U.S. Department of Health and Human Services, Office of the Assistant Secretary for Health, Office of Population Affairs, Rockville, MD USA; 2 Mathematica Policy Research, Princeton, NJ USA

**Keywords:** Sustainability, Office of Adolescent Health, Office of Population Affairs, Pregnancy Assistance Fund, Expectant and parenting teens

## Abstract

**Introduction:**

A common concern of federal funders and grant recipients is how to sustain program activities once their federal funding period ends. Federal funding can be intended to develop or seed a program but not necessarily to continue its activities indefinitely. Understanding the importance of programmatic sustainability, the Office of Population Affairs (OPA) conducted research in 2015 on the elements that contribute to sustainability. As part of the Sustainability Study, OPA collected information from former Pregnancy Assistance Fund (PAF) program grantees.

**Methods:**

Grantees that were awarded cohort 1 PAF program funding (2010–2014) but not awarded cohort 2 funding (2014–2017) were eligible for study inclusion because their OPA funding ended more than 1 year prior to the Sustainability Study, allowing for an assessment of sustainability after federal funding. Seven former PAF grantees were identified as eligible. Interviews were conducted with six of these grantees; grant applications and interim final reports from all seven were reviewed.

**Results:**

Five lessons emerged from interviews and review of grant documentation. Programs successfully continuing beyond the federal grant period tended to (1) diversify funding sources, (2) communicate regularly with key stakeholders, (3) form partnerships with like-minded programs, (4) consider implementing evidence-based interventions, and (5) begin planning for sustainability early.

**Discussion:**

By considering these lessons learned from the research, grantees can be well positioned to continue beyond a federal grant period. The lessons garnered from the Sustainability Study have informed, expanded, and affirmed OPA’s sustainability toolkit, sustainability framework, and technical assistance.

## Significance

Existing research suggests that acknowledging the importance of supportive stakeholders, remaining flexible, and beginning sustainability planning early are key elements that contribute to program sustainability. This article supports this existing research and adds information specifically for programs that serve expectant and parenting youth, a population with unique needs.

## Introduction

Federal funders and recipients of federal grants are both concerned with the sustainability of programs beyond the federal funding period (Floersch 2016). To result in positive health benefits, interventions often require program activities be sustained over time, which is why it is crucial to understand the factors that contribute to long-term sustainability (Schell et al. 2013). To address this concern, the U.S. Department of Health and Human Services Office of Population Affairs (OPA), formerly the Office of Adolescent Health (OAH), includes in its grant programs expectations for sustainability planning and provides resources and technical assistance, such as the *OAH Framework for Program Sustainability* (OAH 2017), to support grantees in their efforts to continue programming after their federal funding period ends. To understand the factors that might contribute to sustainability specifically for OPA-funded programs, OPA launched a study^1^ of a small sample of former Pregnancy Assistance Fund (PAF) grantees in 2015. The goals were to determine whether programming was sustained after the funding period ended and to assess the conditions that contributed to sustainability (Asheer et al. 2017). The study would also serve to inform the Framework for Program Sustainability and efforts to refine it to provide useful guidance to grantees.

OPA administers the PAF program, a competitive grant program established in 2010 that aims to improve the health, educational, social, and economic outcomes of expectant and parenting teens, women, men, and their families. The PAF program awards $25 million annually to states and tribal entities to provide a network of supportive services to expectant and parenting youth, adults, and their families to help them complete high school or post-secondary education and gain access to health care, child care, and other supportive services. The PAF program also offers services for pregnant women who are victims of domestic violence, sexual violence, sexual assault, and stalking. The program supports wraparound services for this population provided either directly or by referral. Since 2010, the PAF program has supported multiple cohorts of grantees. Cohort 1 consisted of grants to 15 states and two tribal entities and occurred over 4 years from 2010 to 2014. OPA has competitively funded four other cohorts to date.

During cohort 1, OPA began systematic efforts to both understand program sustainability and provide technical assistance on sustainability for grantees. Based on a series of discussions, including input from grantee representatives, OPA adopted a common sustainability definition: a sustained program is one in which organizations effectively “leverage partnerships and resources to continue programs, services, and/or strategic activities that result in improvements in the health and well-being of adolescents” (OPA 2019). Although sustainability can have multiple definitions, the definition adopted by OPA takes into account its vision and mission to advance best practices that improve the health and well-being of America’s adolescents and allows flexibility for grantees to shape a vision of sustainability that works best for them (OAH 2017; Scheirer 2005). OPA designed a sustainability toolkit with resources for grantees based on currently available literature, grantee input, and research. The toolkit includes a sustainability framework identifying eight different factors of sustainability (Table 1), a resource guide, and assessment tool (Asheer et al. 2017; OAH 2017, 2019).

**Table 1:** OAH sustainability framework and factors

Original *OAH Framework for Program Sustainability* and factors (2014)	Updated *OAH Framework for Program Sustainability* and factors (2017)
1. Create an action strategy	*Strategize*: create an action strategy
2. Assess the environment	*Assess*: assess the environment
3. Be adaptable	*Lead*: identify, engage, and develop leaders
4. Secure community support	*Evolve*: remain flexible and evolve
5. Integrate programs services into community infrastructure	*Communicate*: communicate with stakeholders
6. Build a leadership team	*Integrate*: integrate programs services into community infrastructure
7. Create strategic partnerships	*Partner*: build strategic partnerships and mobilize the community
8. Secure diverse financial opportunities	*Diversify*: secure diverse financial opportunities

In 2014, OPA made the toolkit available to OPA grantees, including grantees in cohort 1 of the PAF program that were then in their final year of funding (OAH 2014). Sustainability planning was not formally required for cohort 1 PAF program grantees. In 2015, building upon efforts to assist grantees in reaching their sustainability goals, OPA launched the Sustainability Study presented in this article to identify and better understand the elements that contribute to sustainability for OPA grantees. The researchers looked at whether former grantees were able to sustain their programs and, if so, how (Asheer et al. 2017). The findings from this study were made publicly available in 2017 through a brief published on OPA’s website. This article discusses the findings from the Sustainability Study and offers a discussion about how the findings and lessons learned have translated into sustainability technical assistance for OPA grantees. The findings confirm previous results from the existing literature, such as acknowledging the importance of supportive stakeholders, remaining flexible, and beginning sustainability planning early (Ireys et al. 2018; Schell et al. 2013; Scheirer 2005). The study also adds information specifically for programs that serve expectant and parenting youth, a population with unique needs.

## Methods

Grantees that were awarded cohort 1 PAF program funding (2010–2014) but not awarded cohort 2 PAF program funding (2014–2017) were eligible for study inclusion because their OPA funding had ended more than 1 year prior to the Sustainability Study, allowing for an assessment of sustainability after federal funding. Seven of the 15 former cohort 1 PAF grantees were identified as eligible. An email was sent to gather information from eligible former grantees about whether the program had continued beyond PAF grant funding and to invite them to participate in the study. Semi-structured telephone interviews were conducted with six former PAF grantees, lasting roughly 90 minutes each.^2^ One program staff member was interviewed for each site. The interviewees were directly involved with implementation or administration of the program during the PAF grant period and were able to provide insight into what happened to their programs after funding concluded. Prior to the interviews, the study team reviewed grant applications and interim reports from the former PAF grantees to familiarize the interviewers with the projects implemented during the grant period and to assess activities that may have contributed to whether a former grantee had been able to sustain their program (Asheer et al. 2017). Interviews were recorded and transcribed; the team then analyzed the transcripts using Excel to organize the data and elicit patterns and themes responding to the study’s key research questions on factors affecting sustainability. This manuscript is not based upon clinical study or patient data.

OPA also initiated an update to the Sustainability Resources and formed a multidisciplinary team to review and update the current materials. The team consisted of currently funded grantees, experts, and researchers, including those on the Sustainability Study. By involving one of the researchers on the team, lessons learned from the study could be incorporated directly into the update of the resources. While the materials were being updated, the team could cross-reference findings from the study to ensure resources were evidence based.

## Results

Interviews and reviews of interim and final reports revealed the types of programs and services offered and themes across grantees that were eligible for study inclusion. During their grant period, the seven former grantees delivered services including, but not limited to, case management, referrals, educational workshops, medical assessments, counseling services, and incentives. Former grantees served expectant and parenting teens and/or young adults. All grantees relied on subrecipients to deliver some or all programming and were awarded annual funding ranging from $500,000 to $2,000,000. Five of the seven cohort 1 PAF grantees were able to sustain the program in some form after their PAF grant concluded (Table 2).

**Table 2:**
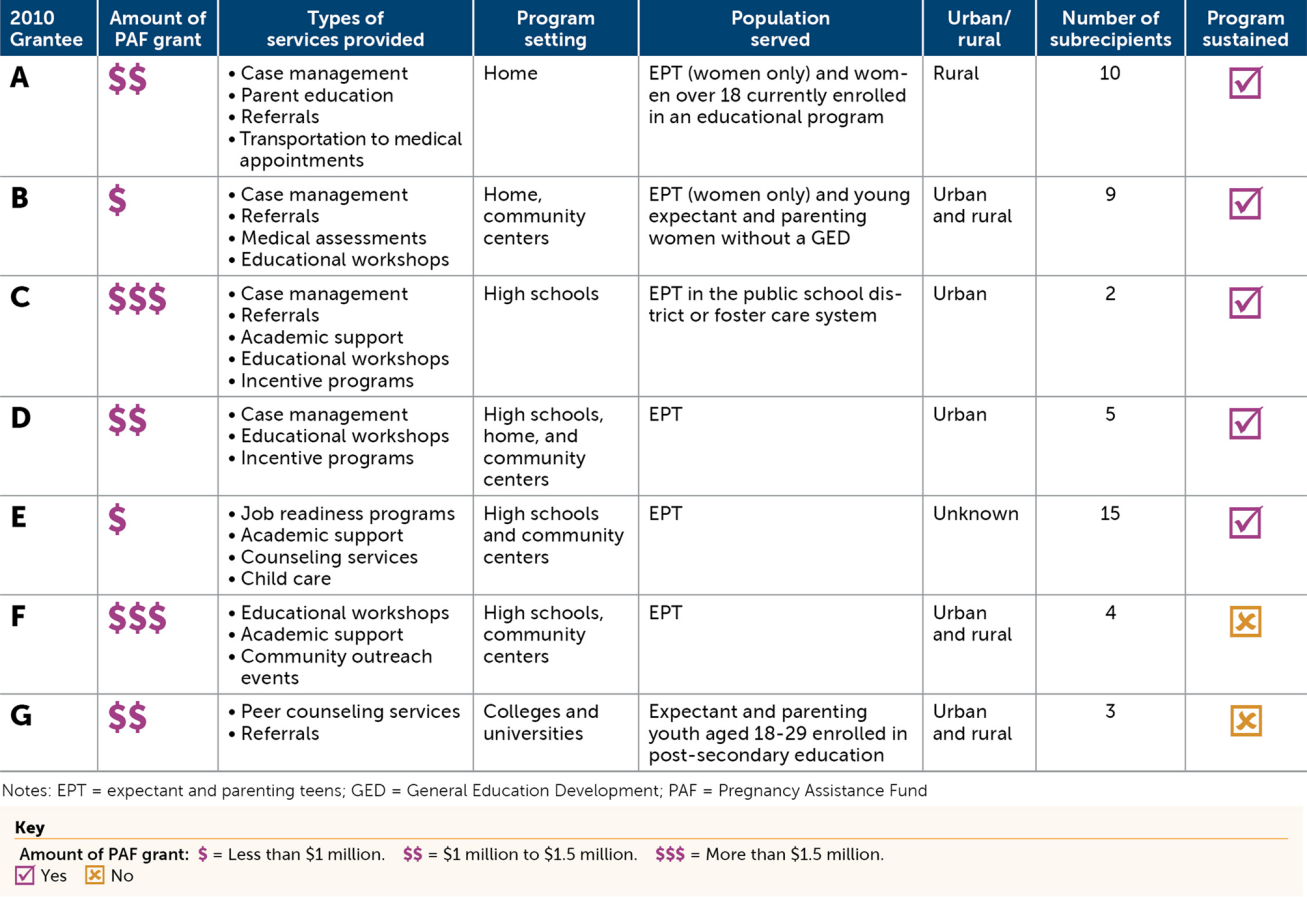
Summary of former PAF grantees (Asheer et al. 2017)

*Note* Taken with permission from Table 1 in Asheer et al. (2017).

Among interview participants, grantees that sustained their programs secured alternative funding support from federal, state, and local governments and/or in-kind funding and adjusted their program structure, reach, and/or services to fit within the new funding streams (Table 3).

**Table 3:**
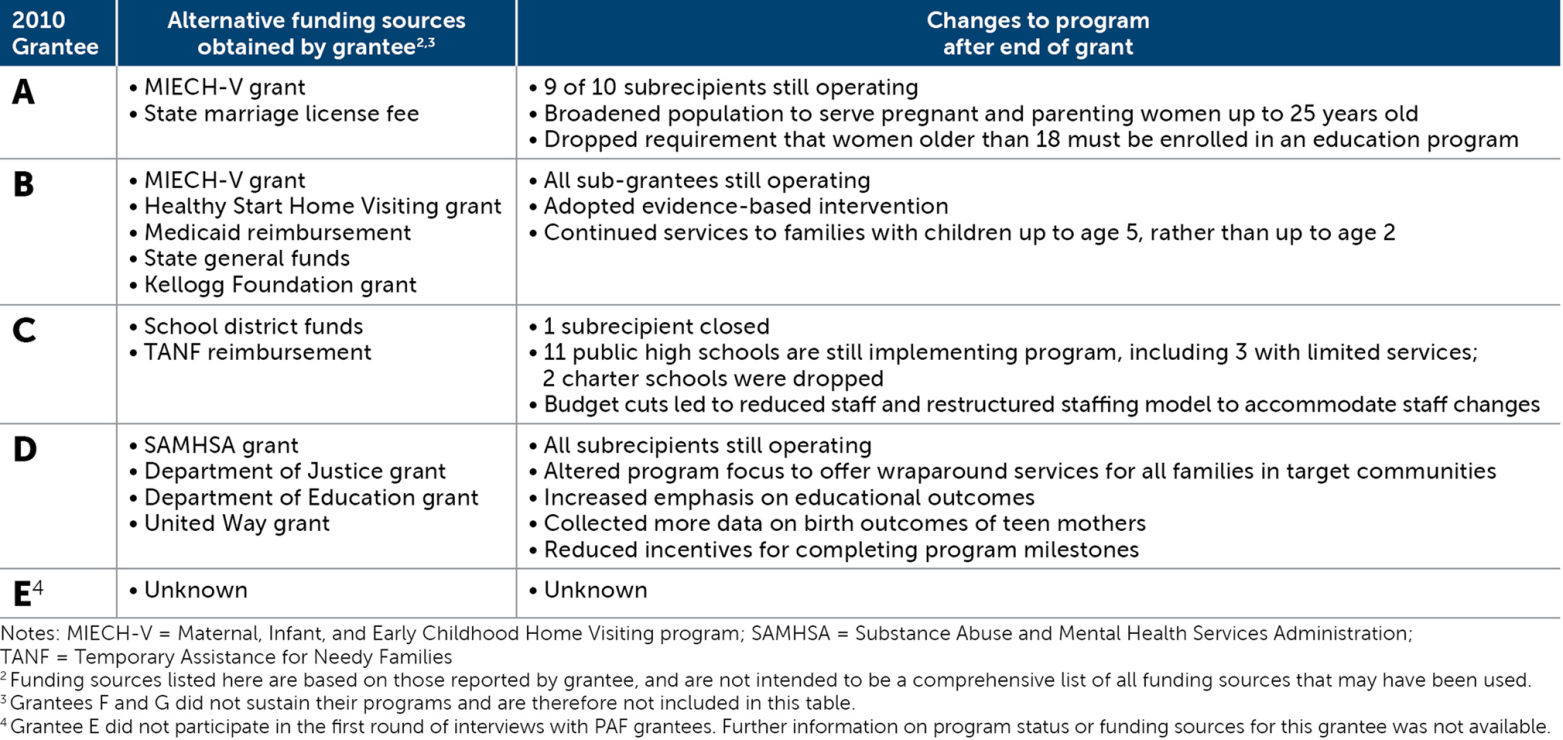
Status of former PAF grantees that sustained their programs (as of August 2016; Asheer et al. 2017)

*Note* Taken with permission from Table 2 in Asheer et al. (2017).

One of the two grantees that did not sustain the program had sought to continue their program but did not secure any new funds to support implementation. The second grantee did not participate in the interviews.

The OPA Sustainability Resources defined eight specific factors associated with action steps leading toward program sustainability. The five overarching lessons emerging from this Sustainability Study largely aligned with some of the key factors identified by the *OAH Framework for Program Sustainability* and helped to further refine and develop the toolkit for grantees. Programs successful in continuing beyond the OPA grant period tended to (1) diversify funding sources, (2) communicate regularly with key stakeholders, (3) form partnerships with like-minded programs, (4) consider implementing evidence-based interventions, and (5) begin planning for sustainability early.

### Lesson 1: Diversify Funding Sources

Identifying and securing continued funding is critical for program sustainability. Former grantees who succeeded in sustaining their programs relied on a combination of federal, state, private, and in-kind support both before and after the grant period concluded. Some federal funding sources that former grantees secured included the Maternal, Infant, and Early Childhood Home Visiting (MIECH-V) program; Healthy Start Home Visiting; Medicaid reimbursement for qualifying services like home visits; Temporary Assistance for Needy Families dollars; and small grants from the Department of Justice (DOJ) and the Substance Abuse and Mental Health Services Administration (SAMHSA). Securing this funding allowed grantees to offer some or even all their services after their PAF grant period ended. In one case, the school district in which the grantee implemented the program absorbed some of the program costs into the district’s budget. In another case, a foundation interested in supporting programs for families with young children in tribal communities approached the grantee with funding to continue operating the program. One state earmarked the fee collected for marriage licenses for the grantee to use toward program costs. No single funding stream was able to support the entire original program, but former OPA grantees combined new grant awards to support different program components.

Former grantees also relied on in-kind support both during the PAF grant program and after funding had concluded. Examples included in-kind staff such as community nurse practitioners, a community health director, and a school district employee. Meeting space for trainings and community events along with parenting supplies offered to program participants were often also in-kind donations. Former grantees emphasized that with multiple funding streams supporting and complementing one another, their programs were more successful than if they relied on one funding stream alone.

### Lesson 2: Communicate Regularly with Key Stakeholders in the Community

Communicating regularly with key stakeholders through focus groups and community meetings helped grantees identify new funding sources, keep programming relevant, and build support for their programs. Strategies included gathering input from participants both to make improvements to the program and to highlight the benefits expressed by participants when presenting to community leaders and potential funders. One former PAF grantee brought program participants to a city council meeting to speak about the positive impact the program had on their lives. Another assembled a team of service providers, youth, and school principals who strongly supported the program to develop a plan for sustainability. Once funding ended, the grantee leveraged those relationships to secure school district funding to continue the program on a slightly smaller scale.

Sustainability was more challenging for two grantees who lacked community and stakeholder support. A shift in state leadership, where buy-in had not been secured, and lack of school district recognition of the program as an academic intervention challenged the sustainability of these programs.

### Lesson 3: Join or Form a Partnership of Like-minded Programs

Establishing formal partnerships with outside organizations increased the likelihood of sustainability. Two of the five grantees that were able to sustain their programs joined coalitions of local organizations doing similar work. Joining these partnerships helped secure other federal funding. One grantee was able to secure an MIECH-V grant awarded to the coalition as a whole. Grantees expressed that coalitions have more success securing grant awards because they have the potential to make a bigger impact than one organization alone and because coalitions allow funders to contribute to multiple entities. Coalitions also allow organizations to work together to strengthen funding applications, as opposed to competing with one another.

Former grantees also established formal partnerships and shared program implementation responsibilities with those partners. Direct involvement in implementation contributed to partner organizations recognizing the importance of the program and becoming champions of the program in the community. Their support improved the grantees’ ability to secure local funding after the PAF grant period had ended.

### Lesson 4: Consider Choosing an Evidence-Based Intervention

The PAF program does not require use of an evidence-based intervention (EBI), and a public evidence review clearinghouse for interventions specifically designed to serve expectant and parenting youth does not exist. Former grantees explained that implementing EBIs benefits organizations for two reasons—to increase the likelihood of securing federal funding and to gain buy-in from local stakeholders. Two former grantees described the benefits associated with implementing programs that had shown positive impacts in an evaluation. One former grantee chose to implement an EBI partway through PAF grant funding in response to an award from the MIECH-V grant program, which required use of an EBI (Health Resources & Services Administration 2019). Another former grantee serving tribal populations noted the Home Visiting Evidence of Effectiveness (HomVEE) review, a federal clearinghouse for home visiting programs, did not include a program designed for tribal populations [Administration for Children and Families (ACF 2016a)]. Once Family Spirit, an intervention designed for pregnant women and families with children younger than age three in Native American communities, was added to the HomVEE review, the grantee began implementing that intervention and attributes doing so as the reason it secured MIECH-V funding after the PAF grant period ended (ACF 2016b). Additionally, implementing an EBI allowed organizations to cite evidence of effectiveness when presenting information to stakeholders to increase their chances of securing alternative funding.

### Lesson 5: Begin Planning for Sustainability as Early as Possible in the Grant Period

To increase the likelihood of sustaining a program, grantees will ideally plan and implement all four lessons presented above well before the grant period ends. One former grantee that sustained its program joined a coalition approximately 1 year after award of PAF funding, and another formed a sustainability team 1 year prior to the conclusion of the grant period.

Grantees that did not begin planning for sustainability well in advance of the end of the grant period faced significant challenges. They experienced long interruptions in service provision or discontinued the program entirely. Grantees that did begin planning early were able to transition their programs—making the shifts required by newly acquired funding streams—with limited interruptions.

## Discussion

Although federal grantees differ, program sustainability is an important consideration for all of them. Applying the information from the Sustainability Study may help position many different types of organizations, such as state and tribal entities, to continue programming once federal funding ends.

The Sustainability Study provides evidence of the importance of securing diverse financial opportunities, a factor in the *OAH Framework for Program Sustainability* (OAH 2017). Grantees who sustained were not only able to leverage foundation and local resources such as school district funding but also to secure federal funding for parts of program activities through the DOJ, SAMHSA, and MIECH-V, among others—likely due, in part, to the intersectional nature of PAF programming. The PAF program seeks to support expectant and parenting young families with access to a variety of education, social, and health services. Funded grantees can leverage other existing sources of funding to support particular service activities. The Sustainability Study showed that communication with diverse stakeholders—another factor in the OAH framework—such as with community leadership or program participants, contributed to successful continued programming. The study also demonstrated that joining a coalition impacted program sustainability and helped projects effectively leverage partnerships and resources. These activities directly align with the OAH framework factor “Build strategic partnerships and mobilize the community” (OAH 2017). Although few evidence-based programs currently address the expectant and parenting youth population, many are in development or under evaluation. The development of evidence-based programs offers an opportunity for future projects working with expectant and parenting young women, men, and their children. Lastly, creating an action strategy was highly important, but the key was to start the process before a grant award or soon after.

These lessons learned from the Sustainability Study affirmed the sustainability technical assistance available to grantees through OPA. Resources including the sustainability toolkit; the *OAH Framework for Program Sustainability*; and technical assistance (e.g., webinars, discussions with experts, and conference presentations) align well with discoveries from the Sustainability Study. The research also indicated areas of opportunity, improvement, and expansion. Accordingly, OPA used the research from the Sustainability Study along with a peer review process to update the OAH sustainability framework (Table 1; OAH 2017). With the lessons learned from the Sustainability Study, these factors help provide a structure for programmatic sustainability among grantees. These lessons have been framed so that they are not specific to programs serving expectant and parenting youth and can be useful to a broader audience of grant recipients. Providing sustainability technical assistance resources based on lessons from the field can be a useful approach for other grantmaking organizations.

Several limitations should be considered. The sample size of the study was small (n = 6) and study participants had previously been awarded grant funding from the same federal program, which means the types of entities receiving funding (states and tribes are eligible for funding through the PAF program), populations served, and types of programs implemented were similar. Additionally, the reported lessons learned are lessons that appeared to be effective based on themes gathered through interviews and grantee reports; however, the effectiveness of these lessons has not been tested. These limitations raise questions about the generalizability of the findings presented. A larger study of the sustainability of programs serving expectant and parenting youth and a systematic review of existing literature are needed. By considering and applying these lessons learned, projects supporting expectant and parenting youth and projects providing support to other populations may be positioned to continue services beyond a federal grant period.

## Footnotes

1 Study was conducted under contract by Mathematica Policy Research.

2 One former grantee was not responsive to requests for an interview.
